# Comparative Study of Statistical Approaches and SNP Panels to Infer Distant Relationships in Forensic Genetics

**DOI:** 10.3390/genes16020114

**Published:** 2025-01-21

**Authors:** Andreas Tillmar, Daniel Kling

**Affiliations:** 1 Department of Forensic Genetics and Forensic Toxicology, National Board of Forensic Medicine, SE-587 58 Linköping, Sweden; andreas.tillmar@rmv.se; 2 Department of Clinical and Experimental Medicine, Faculty of Health Sciences, Linköping University, SE-582 25 Linköping, Sweden; 3 Department of Forensic Sciences, Oslo University Hospital, NO-0450 Oslo, Norway; 4 Department of Biostatistics (BIAS), Norwegian University of Life Sciences, NO-1433 Aas, Norway

**Keywords:** investigative genetic genealogy, SNP, forensic statistics, classification, identity by descent, FORCE, Kintelligence, segment, LR, KING, kinship coefficient

## Abstract

**Background/Objectives**: Inferring genetic relationships based on genetic data has gained an increasing focus in the last years, in particular explained by the rise of forensic investigative genetic genealogy (FIGG) but also the introduction of expanded SNP panels in forensic genetics. A plethora of statistical methods are used throughout publications; in direct-to-consumer (DTC) testing, the shared segment approach is used, in screenings of relationships in medical genetic research, for instance, methods-of-moment estimators, e.g., estimation of the kinship coefficient, are used, and in forensic genetics, the likelihood and the likelihood ratio are commonly used to evaluate the genetic data under competing hypotheses. This current study aims to compare and contrast examples of the aforementioned statistical methods to infer relationships from genetic data. **Methods/Results**: This study includes some historical and some recently published panels of SNP markers to illustrate the strength and caveats of the statistical methods on different marker sets and a selection of pre-defined pairwise relationships, 1st through 7th degree. Extensive simulations are performed and subsequently subsetted based on the marker panels alluded to above. As has been shown in previous research, the likelihood ratio is most powerful, i.e., high correct classifications, when SNP data are sparse, say below 20,000 markers, whereas the windowed kinships and segment approaches are equally powerful when very dense SNP data are available, say >20,000 markers. In between lay approaches using method-of-moments estimators which perform well when the degree of relationship is below four but less so beyond, say, 4th degree relationships. The likelihood ratio is the only method that is easily adapted for non-pairwise tests and therefore has an additional depth not addressed in the current study. We furthermore perform a study of genotyping error rates and their impact on the different statistical methods employed to infer relationships, where the results show that error rates below 1% seem to have low impact across all methods, in particular for errors yielding false heterozygote genotypes.

## 1. Introduction

Inferring relationships is a topic with applications ranging from standard paternity cases [1] to forensic investigative genetic genealogy (FIGG) [2]. In the former application, the established method in forensic genetics relies on the likelihood ratio [1], which is used both in inferences of kinships as well as in forensic criminal casework. In contrast, FIGG commonly employs versions of the segment approach whereby half-identical stretches, that is, at least one allele common identical by state (IBS), between a pair of individual is used to infer shared genomic segments of DNA identical by descent (IBD) [3,4]. The segment approach is generally restricted to pairwise comparison, whereas extension to more than two individuals has been proposed [5]. On the other hand, the likelihood approach opens up for any pedigree with an arbitrary number of typed individuals. In larger medical genetic studies, relying on swift methods not requiring reaching beyond first cousins, so-called methods-of-moments estimators have been the go-to approach [6,7].

Standard practice in forensic genetics is to run a multiplex of STR markers, with the number of markers ranging from 10 to 30, providing sufficient information to resolve first- and second-degree relationships [8,9]. In contrast, going beyond second-degree relationships, e.g., first cousins, necessitates expanding the marker number by either (i) typing more STRs or (ii) using an SNP marker panel [8]. In particular, genetic sequencing has introduced several SNP panels aiming at resolving distant relationships, say first cousins and beyond, where the standard STR panels are insufficient. For instance, Tillmar et al. [10] developed the FORCE panel encompassing roughly 4000 kinship SNPs as well as a number of phenotypic, ancestry and Y-chromosomal markers. Furthermore, Antunes et al. [11] published details on the ForenSeq Kintelligence panel encompassing roughly 10,000 SNP markers with FIGG and extended kinship inference as the intended applications. In Gorden et al. [12], panels encompassing roughly 25,000 and 95,000 SNPs were presented in connection with the identification of ancient historical remains.

All the aforementioned studies focus on the selection of SNPs and provide some details on the performance using either simulations or real data but generally lack a comparison against other published SNP panels or alternative statistical approaches. In addition, few studies include the risk of false inclusions, that is, inferring unrelated individuals as relatives. Kling et al. previously [13] explored and compared three different methods to infer relationships based on dense (>100,000 SNPs) to semi-dense panels (>10,000 SNPs). In op cit, Kling used simulated data to test the performance of some statistical methods but without using published SNP panels.

The current study aims to compare examples of a wider selection of methods to infer relationships with a focus on their performance in terms of sensitivity (true positive rate) and specificity (false positive rate). In particular, we focus on a selection of recently published SNP marker panels and methods pertaining to these panels. The overarching goal is two-fold, first to provide an idea of the performance of different methods to infer relationships, expanding on the study by Kling et al. [13], and secondly to provide recommendations on what method performs better for each genetic marker panel. As the main purpose is to compare and study expanded SNP panels, we focus on distant kinship, first to third cousins, but also include full siblings as well as a smaller SNP panel for comparison.

## 2. Materials and Methods

### 2.1. Genetic Marker Panels

This study will focus on a subset of genetic markers relevant to infer distant relationships, loosely defined as first cousins and beyond but also including full siblings for comparative purposes. SNP markers available on the Global Screening Array (GSA) from Illumina [14], commonly used by direct-to-consumer genetic companies as well as in other screening studies, will be used as a starting point. We expand the GSA panel with markers from (i) the FORCE markers [10], (ii) (ForenSeq) Kintelligence markers [11], and (iii) the Parabon 25 K and 95 K panels presented in Gorden et al. [12]. At the other end of the spectrum, we include the marker from the (ForenSeq) Signature panel [15]. We subsequently exclude markers with more than a single alternative allele, retaining only bi-allelic SNP markers. To mimic the approach taken by Gorden et al. [12], we prune the 25 K/95 K and GSA marker panels, removing markers with linkage disequilibrium (LD). We use a sliding window approach in PLINK with the –pariwise flag [6]. Specifically, we set r^2^ > 0.2, with window size = 100 kb, resulting in a subset of marker passing this pruning procedure, as seen in Table 1. We note that the --pairwisephase, option in PLINK, where a pre-phasing of the data is performed, prunes a considerably greater number of markers, indicating that there may still be LD in the data with the less stringent procedure alluded to previously. However, we use the --pairwise option for reasons of making comparison to previous studies, although we further note that Kling [16] used a third approach, similar to the –pairwisephase option, where the phase information from the 1000 G is used to compute LD between markers for a given population sample. Moreover, the pruning procedure only uses population data from a European population (see Section 2.2), whereas the result from pruning using another population, for instance, African, will possibly be different. Genetic positions, that is, centiMorgans (cm), are obtained from the Rutger’s repository and interpolated for markers with missing positions [17].

### 2.2. Reference Data

Genotype data from the 1000 Genomes project (Phase 3, build 20130502) were download following links from https://www.internationalgenome.org/data (accessed on 18 December 2024) [18]. We extracted individuals with Non-Finnish European (NFE) ancestry (GBR, TSI, CEU, and IBS), yielding in total 404 unrelated individuals. Allele frequencies were generated for the included SNPs using the extracted NFE individuals. In detail, we used bedtools intersect to extract the unison of the markers presented in Table 1 from the 1000G vcf files for each chromosome separately and subsequently merged the results into a single vcf file. Individual files were subsequently created for each panel in Table 1 using a custom R-script where only the NFE individuals were extracted. These files serve as input for the ensuing simulations and comparisons.

### 2.3. Simulation Procedure

We simulate genetic data using methods published by Caballero et al. [19]. In detail, we use the ped-sim script freely available through https://github.com/williamslab/ped-sim (accessed on 18 December 2024). The implementation allows crossover interference [20] to be modeled in the model for pedigree transmission. We use a genetic map published in Bherer et al. [21]. The script simulates whole genome data, i.e., segments, without genotype data as input. In the final step, real haloptype data can be superimposed, yielding as output a vcf file with phased genotypes for the simulated individuals. High-quality phased haplotype data that are freely available are limited, and the 1000 G dataset, referred to previously, will be used to sample founders in each simulation. We perform 10,000 simulations for each relationship, which necessitates an iterative procedure whereby founder haplotypes are drawn and then replaced for each simulation. This will cause a slight bias, in particular since identical rare alleles or individual specific segments can be sampled in two different simulations. Custom R-scripts were used to make iterative calls to ped-sim simulating pairs of full siblings (1st degree), first cousins (3rd degree), second cousins (5th degree), and third cousins (7th degree). In total, 40,000 pairs of relatives were generated. To address unrelated individuals, we compare the 404 individuals encompassing the NFE superpopulation in a pairwise manner, yielding in total 81,407 pairs of unrelated comparisons. Details on scripts for simulations are available from the authors upon request.

### 2.4. Inferring Relationships

We include a total of five different statistical approaches commonly used to infer relationships in various applications. The methods are not exhaustive but represent a selection of approaches traditionally or recently used in inference of relationships. Additionally, we combine the results from the methods to elude if a joint approach can improve the accuracy. The methods are summarized in Table 2 and described in detail next. Only the likelihood ratio, as implemented in the current study, requires formulation of hypotheses, whereas the remaining four are exploratory in the sense that the output provides a value that can be used to infer the most likely relationship pertaining to this value. We also note that only the LR is easily applied when there are more than two individuals involved in the dispute. For outbreed pairwise relationships, we note that these can be described using the three kappa coefficients (*κ*_0_, *κ*_1_, *κ*_2_) denoting the probability that two individuals shared 0, 1, or 2 alleles identical by descent (IBD).

#### 2.4.1. Likelihood Ratio

The likelihood ratio (LR) is the state of the art in forensic applications, providing a weight of the evidence under competing hypotheses [1]. The LR normally compares the genetic data under two mutually exclusive hypotheses, e.g., H1: Individuals P1 and P2 are second cousins versus H2: P1 and P2 are unrelated.

The likelihood requires a mathematical model to be formulated where an algorithm to evaluate the data under a given hypothesis is used. For dense genetic marker data, the Lander–Green algorithm [25], or versions of it, is used. Mostad et al. recently developed an implementation using data from genetic sequencing [26], whereas traditionally, the software Merlin [22] has been used in numerous studies pertaining to inference of relationships. Since our output is simulated genotypes, we use Merlin (version 1.1.2), which provides an efficient way to compute likelihoods for a large number of simulations. We note that our likelihood model does account for genetic linkage but not linkage disequilibrium (LD). The pruning procedure, alluded to previously, is used as a proxy to alleviate linkage disequilibrium. In the calculation, we use both the genetic map described in Section 2.1 and also a fictive genetic map where all markers are positioned 100 cM apart, which roughly translates as treating the markers as independent (unlinked).

#### 2.4.2. Maximum Likelihood Estimate

Korneliussen et al. [23] describe an algorithm to efficiently estimate the 15 Jacquard coefficients that maximize the likelihood of the data for inbreed pairwise relationships. The algorithm has its main merits in that it can account for sequence read/quality data through genotype likelihoods. The algorithm does not per se account for genetic linkage or linkage disequilibrium, although the authors state that the likelihood then turns into a composite likelihood when these are present. We will use the simplified output, referring to non-inbreed pairwise relationships, and only report the kappa values, also known as the Cotterman coefficients, denoting the probability that two individuals share 0, 1, or 2 alleles identical by descent (IBD).

#### 2.4.3. Methods of Moment Estimators

We refer to the approaches published in Manichaikul et al. [7] as methods of moments (MoM) estimators. In particular, we use what the authors refer to as the robust kinship estimate. Briefly, the estimator uses IBS states for a pair of individuals to infer the kinship coefficient (theoretically computed as *κ**_1_***/2 + *κ**_2_***),

$$\phi_{i,j}=\kappa_{1}/2+\kappa_{2}=\frac{N_{Aa,Aa}-2N_{AA,aa}}{2N_{Aa}^{\left(i\right)}}+\frac{1}{2}-\frac{1}{4}\frac{N_{Aa}^{\left(i\right)}+N_{Aa}^{\left(j\right)}}{N_{Aa}^{\left(i\right)}}$$

where the kinship coefficient (*ϕ*_j,j_) for individuals *i* and *j* is estimated from the number of markers where both individuals are heterozygotes (*N_Aa,Aa_*), opposite homozygotes (*N_AA,aa_*), and number of markers where the individuals are heterozygotes *N^(i)^_Aa_* and *N^(j)^_Aa_*, respectively. The equation also requires that individual *i* has higher heterozygosity than individual *j*. For brevity, we refer to the approach as KING, in reference to the implementation by Manichaikul et al. [7].

#### 2.4.4. Segments

An appealing approach in applications where dense genetic SNP data are available is referred to as the segment approach [3,4,27,28], whereby half-identical shared genetic segments are detected and accumulated across the genome for a pair of individuals. The segment method is widely used in genetic genealogy applications where dense SNP data, typically >100,000 markers, are available from direct-to-consumer genetic companies. Although some implementations take a more statistical approach [27], we focus on mimicking the core method. We use a custom implementation of the approach requiring two input parameters to call shared segments as IBD between pairs of individuals, number of SNPs in each segment, and the length of a segment in cM. We use a minimum length of 5 cM to call segments as IBD. We vary the required number of SNPs included in a segment based on the density of the genetic marker panel based on a small optimization study see Supplementary Table S1.

#### 2.4.5. Windowed Kinship

The last approach was published by Snedecor et al. [24], with special attention to the Kintelligence markers, although the approach in itself is not restricted to those markers. Briefly, the model in op cit uses a version of the segment approach tailored for lower density marker panels. In particular, the model uses a sliding window of a certain size to identify seed segments (normally small segments) where two metrics are computed for each window, the kinship coefficient (*a*) and the proportion of markers with at least one shared allele (*f*). The windows are subsequently merged into superwindows and finally called as IBD segments or not based on a and f relating to the IBD status of the superwindow. We use *f* = 0.95 and *a* = 0.23, as suggested by Snedecor et al., and vary the window size according to the size of the genetic marker panel, see Supplementary Table S1, where, for instance, data from the Kintelligence panel are analyzed using a window size of 60 (personal communication with the authors of [24]). The approach to merge smaller windows into larger resembles the approach described in the Ancestry.com white paper for seed segments [29].

### 2.5. Exploring Impact of Errors

We study the impact of errors by inducing errors randomly among the markers for each marker panel separately. We subsequently analyze the data using the methods alluded to in Section 2.4 without accounting for errors and study the impact. In detail, we induce errors randomly into one of the simulated profiles for our pair of individuals using the error rates indicated in Table 3 where we divide the errors into three different types, (i) a homozygote genotype turns heterozygote, (ii) a homozygote genotype turns opposite homozygote, and (iii) a heterozygote genotype turns homozygote. We perform relationship inference using LR, windowed kinship, and the segment approach, exempting KING and ngsRelate, with the same settings as previously indicated, see Supplementary Table S1.

### 2.6. Classification of Relationships

Since only the likelihood approach provides a number entailing the relationship that maximizes the likelihood of the data, our study necessitates a method to classify pairs of individuals into the relationship classes we study. Similar to Kling et al. [13], we classify individuals as full siblings (1st degree), first cousins (3rd degree), second cousins (5th degree), third cousins (7th degree), or unrelated. Some summary statistics are displayed in Table 3 with inference criteria obtained from Manichaikul et al. [7] as well as Kling et al. [13].

For the likelihood approach, we compare the output for each of the relationship classes alluded to previously and report the class with the highest likelihood; that is, the likelihood is computed for each of the aforementioned relationships, regardless of the true underlying relationship. We note that one may use a specific difference threshold, for instance, an LR > 10 in the comparison of the most likely and the next most likely relationship class, which may exclude false positives but also exclude true positives.

For the windowed kinship and the segment approach, both providing total accumulated shared cM, we use data retrieved from https://www.ancestrycdn.com/support/us/2020/08/matchingwhitepaper.pdf (accessed on 18 December 2024), which is based on a large simulation study performed by DTC company Ancestry.com (Lehi, UT, USA). Briefly the data are based on a table of the most likely relationship class based on a total shared cM value (See Figure 5.2 in the aforementioned white paper). As mentioned, data are based on simulations; however, we find that the results align well with our simulated data). In this context, we also make the notion that DTC generally does not make an exact inference of a degree of relationship but rather gives a range, say first cousins—second cousins, to incorporate the overlap of total shared cM for different degrees. In addition, we classify relatives based on the logistic regression model used in Kling et al. [13].

Furthermore, we classify individuals by summarizing the number of shared segments and the average length of shared segments by comparing output from the R library ibdsim2 (https://github.com/magnusdv/ibdsim2, accessed on 18 December 2024). In detail, we compute the minimum Euclidean distance to mean output generated through 10,000 simulations in ibdsim2. Briefly, and similar to the data generation by ped-sim, ibdsim2 generates genomic sharing given a genetic map (we use the decode map included in the library) with a chi-square crossover model, sex-specific map turned off, and cutoff equal to 5. We summarize the average length and total segments for each relationship class included in our study. We subsequently compute the Euclidean distance for each of our simulations and report the relationship class that minimizes the distance. This approach could potentially separate relationships belonging to the same degree, e.g., half siblings and uncle/nephew.

To infer relationship class based on the method-of-moments results, we use thresholds first presented in Manichaikul et al. [7] and later expanded on in Kling et al. [13]. We also use the logistic regression model presented in Kling et al. [13] for inference of the degree of relationship (1–5) based on the estimated kinship coefficient.

Finally, the output from ngsRelate is the Jacquard coefficient that maximizes the likelihood of the data [23]. We use the condensed kappa coefficients and compare the output with expectations for each relationship class (see Table 4) by computing the Euclidean distance and select the class that minimizes this distance.

## 3. Results

The results are summarized using classification rates, measured through the rate of correct/incorrect classifications for each degree of the investigated relationship. We note that in exploratory applications, correctly establishing the exact degree of relationship may not always be crucial, although we argue that the best way to compare the different methods is through classifications.

### 3.1. True IBD Sharing

**Figure 1:**
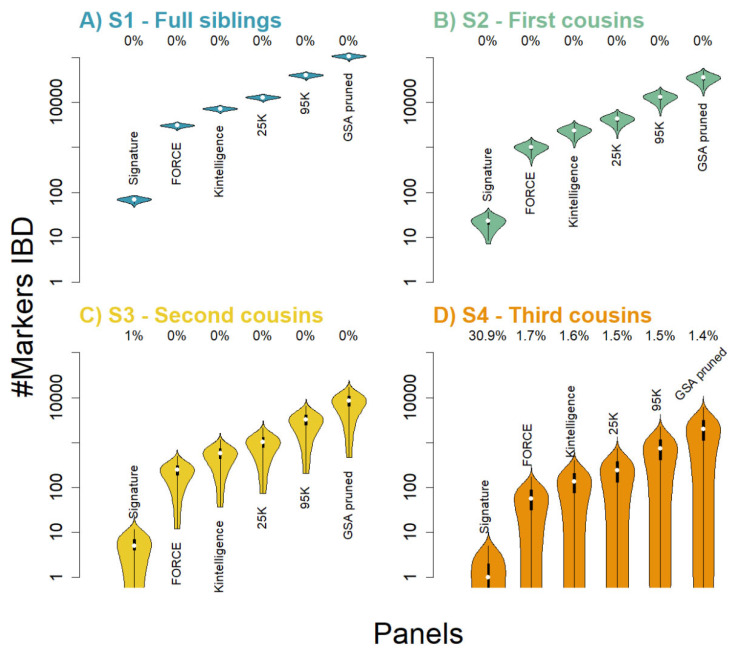
Total number of markers IBD across the 10,000 simulated pairs of relatives for the different relationships: full siblings through third cousins. Different marker panels are displayed across the x-axis, with the y-axis displaying total number of SNPs in true IBD status. Displayed above each violin plot is the percentage of pairs of simulated relatives where zero SNPs are IBD. (**A**) full siblings (teal), (**B**) first cousins (green), (**C**) second cousins (yellow) and (**D**) third cousins (orange).

In addition to genotypes, each simulation produces true IBD sharing as a separate file, given as chromosomal coordinates (both physical and genetic) for each true IBD segment and for each relationship. The results are summarized in Supplementary Figure S1, where the distribution of total segment sharing is visualized. For each marker panel, we further intersected the output chromosomal coordinates, that is, true IBD segments, with the simulated data for each relationship separately. Figure 1 displays the total number of markers located in IBD segments for each panel and summarized across the 10,000 simulations, which illustrates, as expected, that the Signature panel is not suited for distant relationships with few IBD SNPs. In other words, the SNPs located in IBD segments are the truly kinship informative markers and consequently provide information on the relationship. Furthermore, we note that for roughly 1.5% of the third cousins, none of the included panels will include true IBD SNPs, either because (i) the third cousins are genetically unrelated or (ii) true IBD regions are not covered by the SNPs.

### 3.2. Classifications

**Figure 2:**
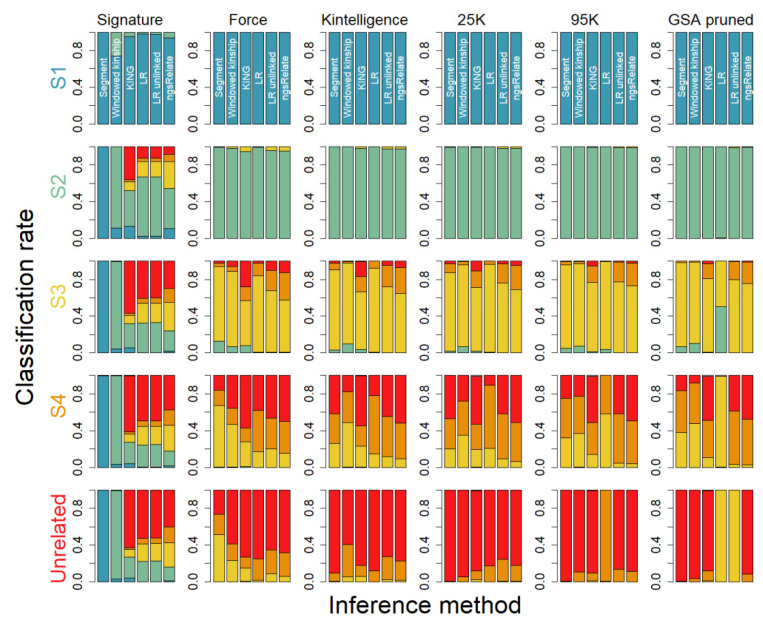
Classification of relatives based on 10,000 pairs of simulated relatives including S1 = full siblings (teal), S2 = first cousins (green), S3 = second cousins (yellow), S4 = third cousins (orange), and >80,000 pairs of unrelated (red). Each row in the figure represents the true relationship and each column a specific SNP panel. Classification is performed using 6 different methods, detailed in the main text, including (from left to right) Segment, Windowed kinship, KING, LR, LR_unlinked, ngsRelate, with the colors representing the classification.

Classifications are performed such that each classification results in a number in the range 1–5, where 5 signifies unrelated and 1 full siblings. We summarize the number of correct classifications indicating that, for instance, a class 1 pair of relatives (full siblings) are classified as full siblings. We note that in a genetic genealogy investigation, classifying a pair of individuals as first cousins instead of second cousins may not be detrimental but does carry a certain cost. A summary of the results is illustrated in Figure 2, showing that (i) full siblings is readily distinguished with all methods across all panels, except for the Signature panel, where the windowed kinship approach displays a slightly lower classification success, (ii) first cousins are distinguished using all methods and across all panels to at least a 95% rate (except the Signature panel), (iii) second cousins display varying degrees of classification success with, for instance, the LR method showing the highest rates for FORCE, Kintelligence, and the 25 K/95 K panels, but also high rates using the windowed kinship approach across all panels (except the Signature panel), (iv third cousins display a high degree of variation across the panels and methods, with the LR approach showing the highest success rate across the mid-sized panels (FORCE, Kintelligence, and 25 K) and windowed kinship and segment approaching showing the highest success for the larger panels (95 K and GSA pruned), and (v) unrelated are distinguished at fairly high rates (>80%) for the FORCE/Kintelligence panels for all methods except the segment/windowed kinship, showing inflated false positive values and for the denser panels (95 K and GSA pruned) an increasingly high false positive rate. Supplementary Figures S2 and S3 further illustrate that using average segment length and number from the segment approach has limited added information compared to accumulated segment length and even performs much worse for the less dense marker panels. We note that at some point, perhaps for marker number above say 20,000, the LR approach starts to induce false relatedness, e.g., relatives or unrelated appear more closely related. Particularly interesting is that when not accounting for linkage, this effect seems to be mitigated, to some extent, see, for instance, Figure 2 and the GSA pruned marker panel and classifications of third cousins (S4).

**Figure 3:**
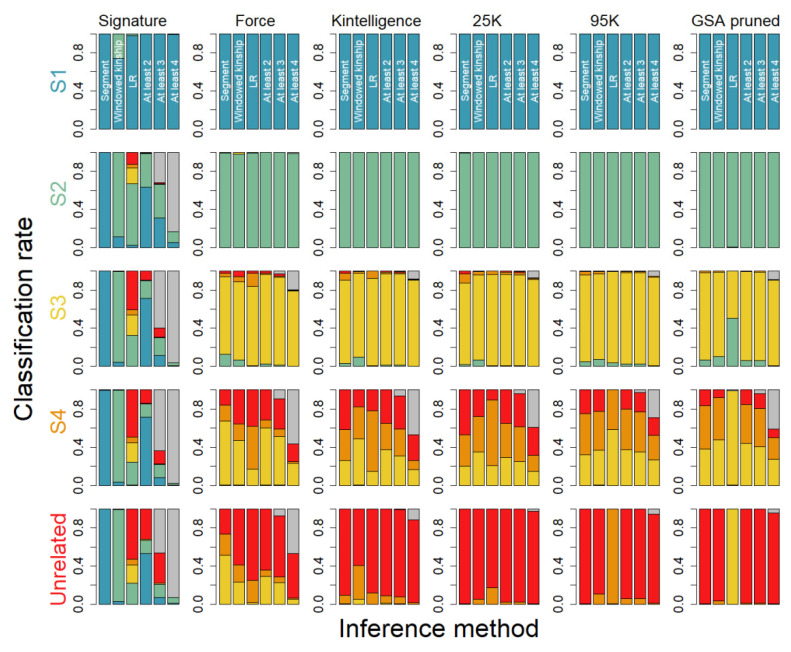
Classification of relatives based on 10,000 pairs of simulated relatives including S1 = full siblings (teal), S2 = first cousins (green), S3 = second cousins (yellow), S4 = third cousins (orange), and >80,000 pairs of unrelated (red). Each row in the figure represents the true relationship and each column a specific SNP panel. Classification is performed using three different methods detailed in the main text including (from left to right) Segment, Windowed kinship and LR as well as joint approaches requiring at least 2, 3, or 4 methods to agree, with the colors representing the classification and grey indicating inconclusive classification.

Figure 3 displays classifications using a combined approach where at least 2, 3, or 4 of the methods need to agree to make a classification. The results, including an inconclusive classification if the methods disagree, illustrates that there is little to gain from a joint approach to relationship inference in comparison to using them separately.

### 3.3. Impact of Errors

**Figure 4:**
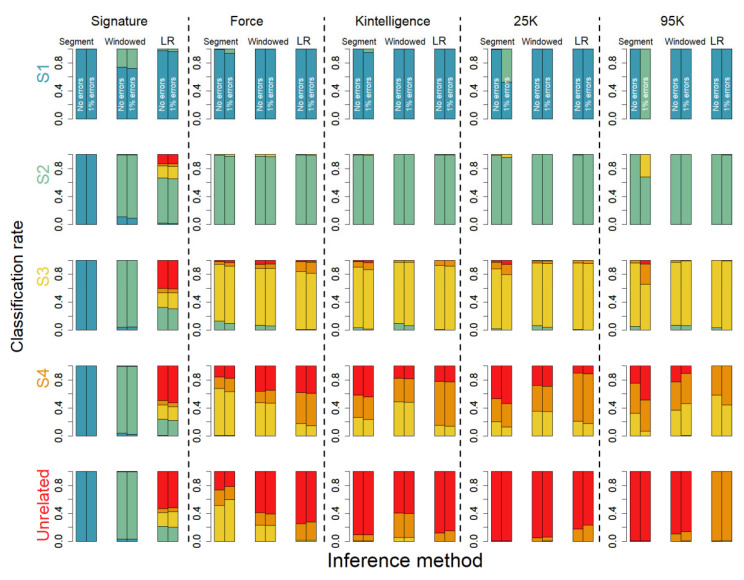
Classification of relatives based on 10,000 pairs of simulated relatives including S1 = full siblings (teal), S2 = first cousins (green), S3 = second cousins (yellow), S4 = third cousins (orange), and >80,000 pairs of unrelated (red). Each row in the figure represents the true relationship and each column a specific SNP panel. Classification is performed using three different methods, detailed in the main text, with the colors representing the classification. Results are displayed for data without and with errors (1% homozygote->homozygote errors).

Figure 4 displays a summary of data with 1% errors (homozygote->homozygote), indicating that there is overall a limited impact with the LR method, whereas, in particular, the segment approach is sensitive, as expected, to false homozygous genotypes, although we note that for most of the included panels it has limited impact. We note that errors pertaining to generating false heterozygotes have no impact on the segment approach since it only potentially generates false IBD segments, but from Supplementary Figures S4 and S5, we can deduce that this has little impact on relationship classification.

## 4. Discussion

Our study describes an extensive simulation approach whereby data are first generated using a genome-wide approach and then subsetted using a selection of published genetic marker panels and analyzed with different statistical inference approaches Our study builds on previous works by Kling et al. [13] studying classification of pairwise relatives for various statistical methods, Mo et al. [30] and Kling [16] studying the performance of the LR method in inference of kinship, Turner et al. [31] studying the impact of genotyping errors for the segment and KING approaches, as well as Woerner et al. [32] studying and classifying relatives from low-coverage sequencing data.

We find that for smaller marker panels, say below 100 markers, the LR approach is the only approach to provide satisfactory classification rates, and the inference is limited to full siblings and to some extent first cousins (Figure 2). For mid-sized panels, say below 20,000 markers, the LR approach is superior to all the other included approaches, in particular for third cousins, where it outperforms the other inference methods. The results also illustrate that the windowed kinship and the segment approach perform well for relationships below third cousins for the mid-sized panels. For very large panels, say above 20,000 markers, the segment approach and the method-of-moment approach grow more useful, necessitating denser data to accurately call segments, in particular, for relationships beyond second cousins, where shorter IBD segments are expected. The windowed kinship approach, suggested by Snedecor et al. [24] for mid-sized panels to detect shared IBD segments, seems to perform on equal terms with the standard segment approach across all marker panels but shows a slight elevation of degree of relationships for the FORCE and Kintelligence marker panels. We further note that when the panel size exceeds, say, 20,000, the LR method starts to cause an inflation of relatives (increasing the degree of relatedness), which we argue is a consequence of the low-stringent approach used to reduce LD in the data. Using a higher stringency approach, such as the ones proposed by Kling et al. [16], would likely reduce the inflated degree of relatedness. Interestingly, not accounting for linkage seems to alleviate this effect to some degree (Figure 2). It has been suggested that using average segment length and segment number could potentially improve the relationship inference instead of using the accumulated segment length (Supplementary Figure S2). However, our data do not suggest that this is useful for the relationship studied; in fact, it performs worse for some panels and relationships, although we acknowledge that including relationships with the same degree, e.g., half siblings and uncle/nephew, would be insolvable using accumulated length alone.

We further performed a small study on the impact of errors on a subset of our data, indicating that errors where homozygote genotypes turn into opposite homozygotes have the highest impact (Figure 4 and Supplementary Figures S4 and S5), in particular for the segment approach. In fact, the LR and the windowed kinship approaches are largely insensitive to the range of errors tested in the current study. We note that the windowed kinship approach does have a slack in the default parameter setting, allowing a certain degree of errors, whereas our implementation of the segment approach does not, although at the cost of classifying relatives closer than they are (see Figure 2).

Our study is restricted at the lower end to full siblings and at the higher degree to third cousins. We believe this is a relevant range of degrees, where full siblings usually can be resolved with STRs alone and where third cousins is the limit of several of the panels of markers or statistical methods. In fact, a considerable proportion of fourth cousins share no genomic IBD segments, in particular if a minimum 5 cM threshold is used (data obtained from https://magnusdv.shinyapps.io/ibdsim2-shiny/, accessed on 18 December 2024).

Finally, we note two limitations with our study. First, a caveat with our model for simulating data is that it re-uses founder haplotypes in each simulation. However, we argue that the bias caused by this is small compared to not using real genotype/haplotype data and also to the variation caused by various settings in the statistical approaches employed. Using founder data from larger studies, such as the UK Biobank (encompassing >500,000 individuals) might prove useful in future studies assessing the performance of methods to infer relationships [33]. Second, we include a limited set of pairwise relationships (two common founders), and the inclusion of half-relationships (a single common founder) or relationships such as first cousins once removed could potentially change the classifications. However, we argue that this would dilute the results into more fine-grained classifications, which, in turn, would cause less comprehensive interpretations. The marker pruning procedure employed in this study uses a low-stringent approach to remove redundant information caused by population LD, primarily since previous studies use such pruning [12] but also as our LR approach is incompatible with LD. Alternative approaches [16] use phasing information to exclude markers, which we also recommend if using the LR approach. The pruning is not per se necessary for calling IBD segments since this method is not impacted by marker redundancy. However, as our pruning is low-stringent, we do believe it has limited impact, as the markers we retain have a high degree of redundant information with the ones we exclude.

## 5. Conclusions

The current study provides a comprehensive comparison of different statistical methods to infer pairwise relationships using a selection of published SNP panels. The results suggest that the choice of statistical methods for relationship estimation must be tailored to the density of SNP data and the degree of relationships being examined. We strongly recommend also assessing the risk of falsely classifying a pair of unrelated individuals as relatives, particularly in scenarios where low-density SNP data or method-of-moments estimators are used. In the context of FIGG and direct-to-consumer genetics, the exact methods and filters used are often proprietary or not standardized, which can lead to variability in performance and error rates. This variability, coupled with the potential for genotype errors, underscores the importance of studying how such errors impact relationship inference accuracy.

## Figures and Tables

**Table 1:** Description of genetic marker panels evaluated in this study. The applications listed are merely examples of the most common applications.

	Signature	FORCE	Kintelligence	25 K	95 K	GSA	Total ^3^
Raw #markers ^1^	150	5422	10,230	24,999	94,723	654,027	664,327
Filtered #markers ^2^	92	4073	9618	17,231	53,593	142,350	202,470
Reference	Jäger et al. [15]	Tillmar et al. [10]	Antunes et al. [11]	Gorden et al. [12]	Gorden et al. [12]	Russell et al. [14]	
Application	Kinship	Kinship	Kinship/Genealogy	Kinship	Kinship	Medical/Genealogy	

^1^ Raw number of markers included in the panel in the given reference. ^2^ Only autosomal markers are used with data available from the 1000 G project. Also LD-based pruning is performed for the 25 K, 95 K, and GSA panels. ^3^ Joint number of markers in all panels.

**Table 2:** Overview of the inference methods used in the current study with some properties for each approach. NN = not needed. LR = likelihood ratio, ML = maximum likelihood.

	LR	ngsRelate (ML)	Methods of Moment	Segment	Windowed Kinship
Genetic linkage	Yes	No	NN	NN	NN
Linkage disequilibrium	No/Yes	No	NN/No	NN	NN
Hypothesis	Yes	No	No	No	No
Allele frequencies	Yes	Yes	No	No	No/Yes
Lowest marker number ^1^	1000	1000	10,000	4000	4000
Application	General relationship inference	Finding most likely Jacquard coefficients	Screening studies	Genetic genealogy	Genetic genealogy
Reference	Abecasis et al. [22]	Korneliussen et al. [23]	Manichaikul et al. [7]	Browning et al. [4]	Snedecor et al. [24].
	LR	ngsRelate (ML)	Methods of Moment	Segment	Windowed kinship

^1^ To solve a case of first cousins. Rough estimate from simulations in this study.

**Table 3:** Error rates used to induce genotype errors in the simulated genetic data.

Hom->Het	Hom->Hom	Hom->Het
0.02	0	0
0.005	0	0
0	0	0.005
0	0	0.02
0	0.001	0
0	0.01	0

**Table 4:** Overview of the relationship classes used in this study with some statistics for each class. The class of unrelated individuals refers to random pairs drawn from a population sample and may or may not contain background relatedness.

Relationship Class	Degree	IBD (κ_0_, κ_1_, κ_2_)	Shared Segment ^1^	MoM ^2^
Full siblings (S1)	1st	0.25, 0.5, 0.25	2460 cM	0.25
First cousins (S2)	3rd	0.75, 0.25, 0	818 cM	0.063
Second cousins (S3)	5th	0.9375, 0.0625, 0	298 cM	0.016
Third cousins (S4)	7th	0.97, 0.0312, 0	49 cM	0.004
Unrelated (Un)	>7th	1, 0, 0	10 cM ^3^	0

^1^ Data obtained from the simulations. ^2^ Data obtained and expanded based on Manichaikul et al. [7]. ^3^ Unrelated will share certain smaller segments by chance.

## Data Availability

Data and scripts to generate and analyze the results can be obtained from the corresponding author upon request.

## Supplementary Materials

The following supporting information can be downloaded at: https://www.mdpi.com/article/10.3390/genes16020114/s1, Table S1: Inference criteria; Figure S1: Distribution of shared IBD segments; Figure S2: Inference of relationships by panel; Figure S3: Inference of relationships by method; Figure S4: Impact of errors by relationship; Figure S5: Impact of errors by method.

## Abbreviations

The following abbreviations are used in this manuscript:

FIGG	Forensic Investigative Genetic Genealogy
NFE	Non-Finnish Europeans
SNP	Single Nucleotide Polymorphism
DTC	Direct-to-Consumer
LD	Linkage Disequilibrium
