# Supplementary material for: Comparative Study of Statistical Approaches and SNP Panels to Infer Distant Relationships in Forensic Genetics
Source: Genes (Basel). 2025 Jan 21;16(2):114. doi: 10.3390/genes16020114 (PMC11855180; doi:10.3390/genes16020114)
Supplement: Supplementary file 1 [file genes-16-00114-s001.zip › genes-3407077-supplementary.pdf]

Supplementary data to "Comparative study of statistical approaches and SNP panels to infer distant relationships in forensic genetics"

Table of contents

1 Supplementary Table S1 – Inference criteria 2
2 Supplementary Figure S1 – Distribution of shared IBD segments 3
3 Supplementary Figure S2 – Inference of relationships by panel........ 4
4 Supplementary Figure S3 – Inference of relationships by method........ 5
5 Supplementary Figure S4 – Impact of errors by relationship 6
6 Supplementary Figure S5– Impact of errors by method........ 7

1 Supplementary Table S1 – Inference criteria

Supplementary Table S1. Summary of settings used to make relationship inference for the different methods detailed in the manuscript and for the different marker panels. Note that only the Windowed kinship and the segment approach requires tuning of parameters used.

	Signature	FORCE	Kintelligence	25K	95K	GSA pruned	Comment
**LR**	NA	NA	NA	NA	NA	NA	Allele frequencies from NFE individuals used.
**Windowed kinship**	Window size=2	Window size=50	Window size=60	Window size=100	Window size=150	Window size=250	Allele frequencies from NFE individuals used. Threshold for kinship coefficient (a=0.22), IBS1 (f=0.95) and minimum segment length (5 cM) same across all panels.
**Segment approach**	#SNPs=2	#SNPs=50	#SNPs=75	#SNPs=125	#SNPs=250	#SNPs=300	Minimum segment length (5 cM) same across all panels.
**ngsRelate**	NA	NA	NA	NA	NA	NA	Allele frequencies from NFE individuals used.
**KING**	NA	NA	NA	NA	NA	NA	No parameters is necessary

2 Supplementary Figure S1 – Distribution of shared IBD segments

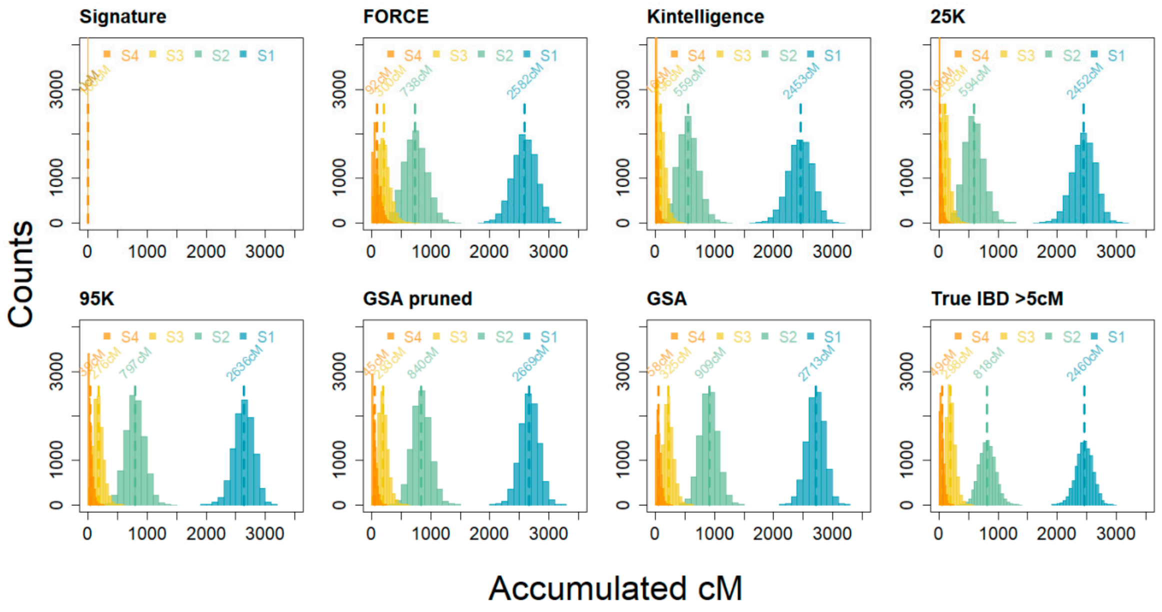

Supplementary Figure S1. Distribution of accumulated shared cM based on 10,000 simulated relatives (detailed in main text). The means for each relationship is displayed above each histogram. The panels are listed as titles with the "True IBD >5cM" indicating the true genome-wide sharing based on the output from the simulations (ped-sim). The figure illustrates that calling IBD segments work well for all panels, on average, even the smaller ones (FORCE and KIntelligence). However, as detailed in the main manuscript, there is a caveat to this performance when investigating the details of the more distant relationships.

3 Supplementary Figure S2 – Inference of relationships by panel

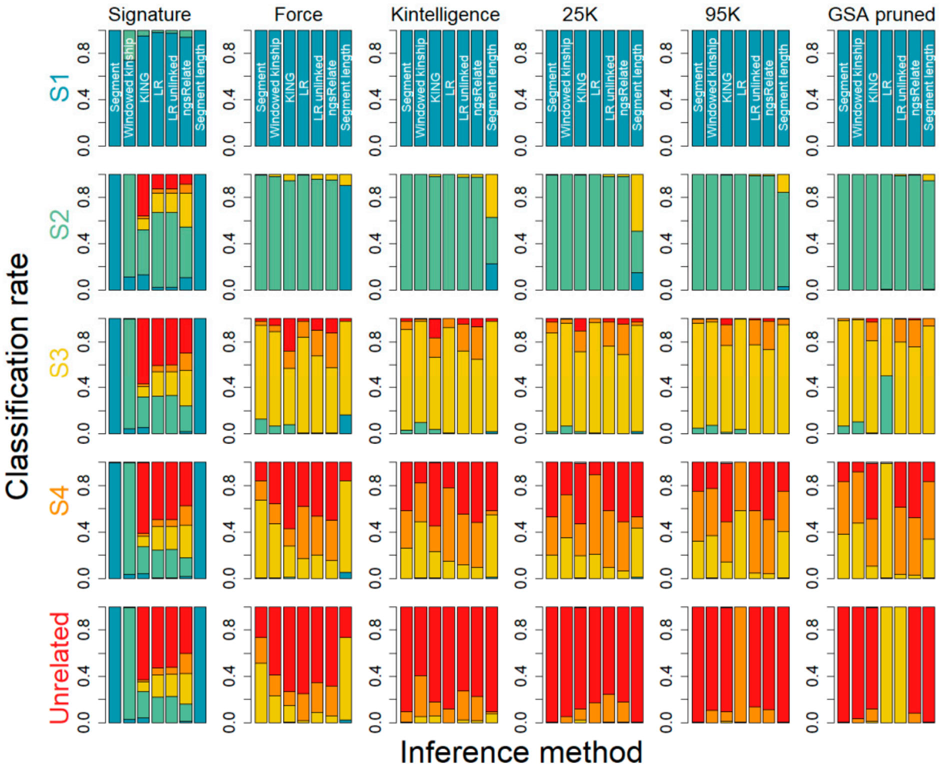

Supplementary Figure S2. Classification of relatives based on 10,000 pairs of simulated relatives including S1=Full siblings (teal), S2=First cousins (green), S3=Second cousins (yellow), S4=Third cousins (orange) and >80,000 pairs of Unrelated (red). Each row in the figure represents the true relationship and each column a specific SNP panel. Classification is performed using 7 different methods, detailed in the main text including (from left to right) *Segment, Windowed kinship, KING, LR, LR_unlinked, ngsRelate and Segment_length*, with the colors representing the classification. Segment_length represents the method whereby the average segment length (in cM) and their number is summarized.

4 Supplementary Figure S3 – Inference of relationships by method

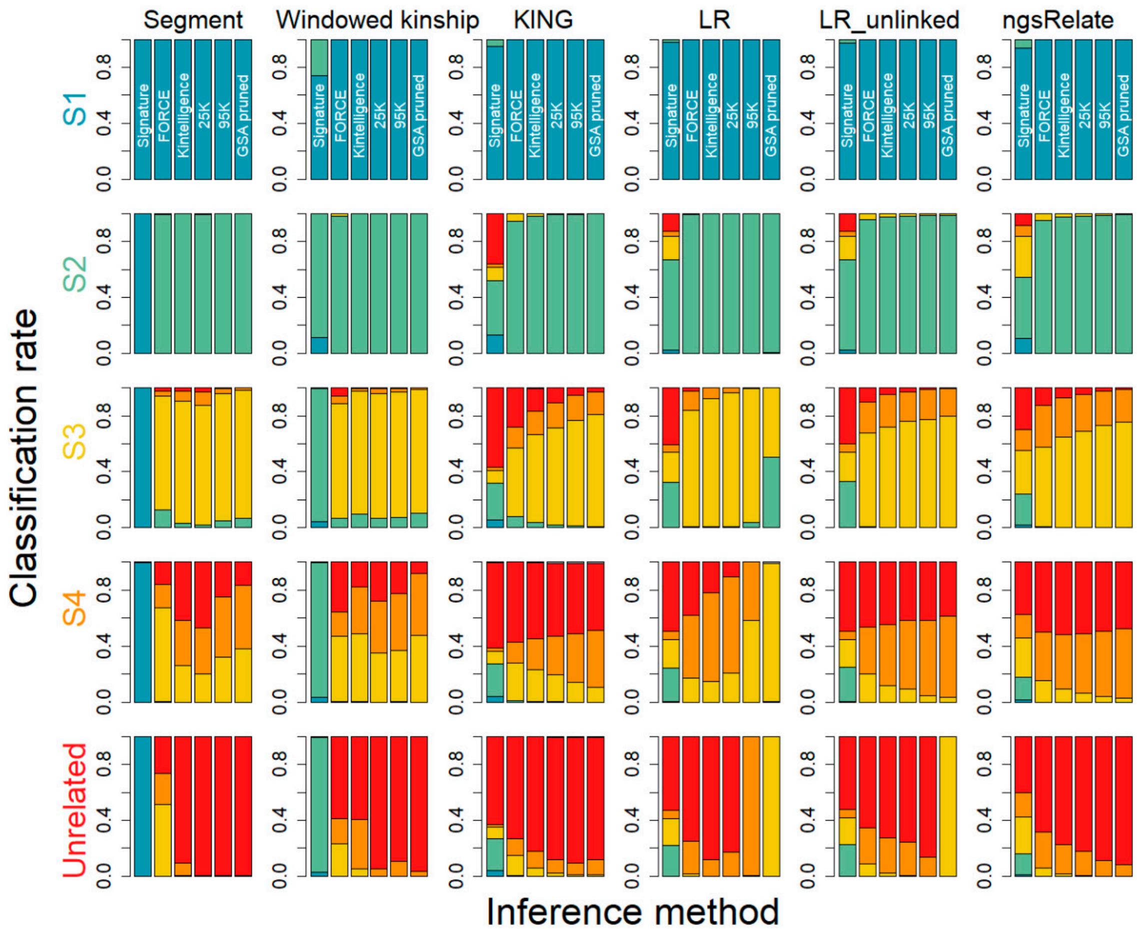

Supplementary Figure S3. Classification of relatives based on 10,000 pairs of simulated relatives including S1=Full siblings (teal), S2=First cousins (green), S3=Second cousins (yellow), S4=Third cousins (orange) and >80,000 pairs of Unrelated (red). Each row in the figure represents the true relationship and each column a specific method used to make relationship inference. Each bar represents a specific panel of SNPs (detailed in the main manuscript).

5 Supplementary Figure S4 – Impact of errors by relationship

The figure below describes a summary of the results where errors are introduced (see data sets on the x-axes). The figure describes a subset of the method used in this study (LR, Windowed kinship and Segment approach).

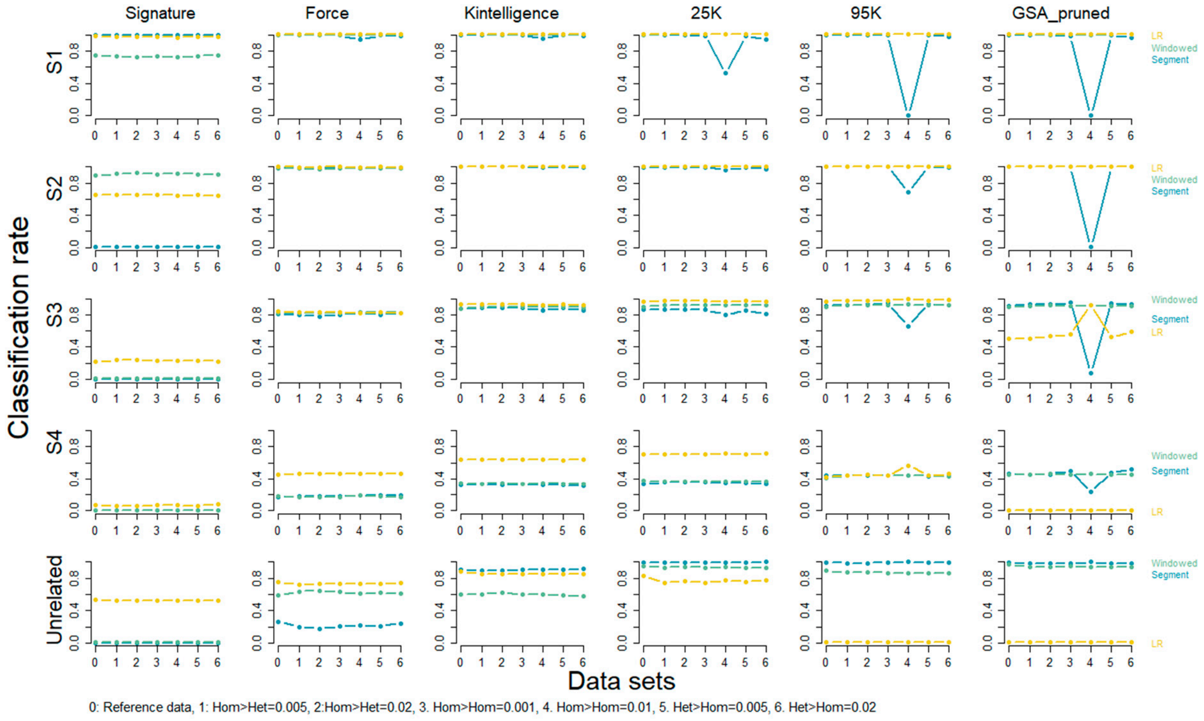

Supplementary Figure S4. Classification of relatives based on 1,000 pairs of simulated relatives including S1=Full siblings, S2=First cousins, S3=Second cousins, S4=Third cousins and 1,000 pairs of Unrelated. Each row in the figure represents the true relationship and each column a specific SNP panel. Classification is performed using three different methods, detailed in the main text including and with different degrees of errors induced in the data (see X-axis and legend below figure). The figure displays the true classification rate, that is the accuracy.

6 Supplementary Figure S5– Impact of errors by method

The figure below describes a summary of the results where errors are introduced (see data sets on the x-axes). The figure describes a subset of the method used in this study (LR, Windowed kinship and Segment approach).

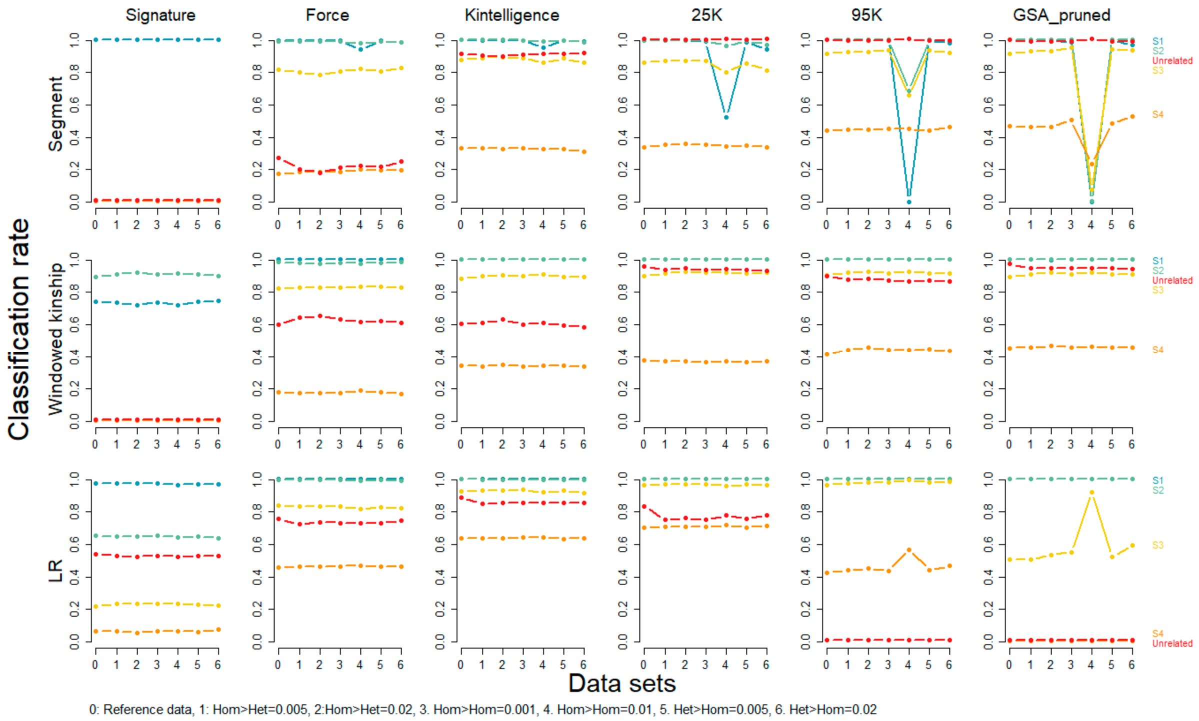

Supplementary Figure S5. Classification of relatives based on 1,000 pairs of simulated relatives including S1=Full siblings (teal), S2=First cousins (green), S3=Second cousins (yellow), S4=Third cousins (orange) and 1,000 pairs of Unrelated (red). Each row in the figure represents an inference method (detailed in the main manuscript) and each column a specific SNP panel. Different degrees of errors are induced in the data (see X-axis and legend below figure). The figure displays the true classification rate, that is the accuracy.
